# High-content screening of Thai medicinal plants reveals *Boesenbergia rotunda* extract and its component Panduratin A as anti-SARS-CoV-2 agents

**DOI:** 10.1038/s41598-020-77003-3

**Published:** 2020-11-17

**Authors:** Phongthon Kanjanasirirat, Ampa Suksatu, Suwimon Manopwisedjaroen, Bamroong Munyoo, Patoomratana Tuchinda, Kedchin Jearawuttanakul, Sawinee Seemakhan, Sitthivut Charoensutthivarakul, Patompon Wongtrakoongate, Noppawan Rangkasenee, Supaporn Pitiporn, Neti Waranuch, Napason Chabang, Phisit Khemawoot, Khanit Sa-ngiamsuntorn, Yongyut Pewkliang, Piyanoot Thongsri, Somchai Chutipongtanate, Suradej Hongeng, Suparerk Borwornpinyo, Arunee Thitithanyanont

**Affiliations:** 1 Excellence Center for Drug Discovery (ECDD), Faculty of Science, Mahidol University, Bangkok, 10400 Thailand; 2 Department of Microbiology, Faculty of Science, Mahidol University, Bangkok, 10400 Thailand; 3 Department of Chemistry, Faculty of Science, Mahidol University, Bangkok, 10400 Thailand; 4 School of Bioinnovation and Bio-Based Product Intelligence, Faculty of Science, Mahidol University, Bangkok, 10400 Thailand; 5 Department of Biochemistry, Faculty of Science, Mahidol University, Bangkok, 10400 Thailand; 6 Department of Biotechnology, Faculty of Science, Mahidol University, Bangkok, 10400 Thailand; 7 Chao Phya Abhaibhubejhr Hospital, Prachin Buri, 25000 Thailand; 8 Department of Pharmaceutical Technology, Faculty of Pharmaceutical Sciences, Naresuan University, Phitsanulok, 65000 Thailand; 9 Center for Neuroscience, Faculty of Science, Mahidol University, Bangkok, 10400 Thailand; 10 Chakri Naruebodindra Medical Institute, Faculty of Medicine Ramathibodi Hospital, Mahidol University, Samutprakarn, 10540 Thailand; 11 Department of Biochemistry, Faculty of Pharmacy, Mahidol University, Bangkok, 10400 Thailand; 12 Section for Translational Medicine, Faculty of Medicine Ramathibodi Hospital, Mahidol University, Bangkok, 10400 Thailand; 13 Department of Pediatrics, Faculty of Medicine Ramathibodi Hospital, Mahidol University, Bangkok, 10400 Thailand

**Keywords:** Drug discovery, Microbiology

## Abstract

Since December 2019, the emergence of severe acute respiratory syndrome coronavirus-2 (SARS-CoV-2) has caused severe pneumonia, a disease named COVID-19, that became pandemic and created an acute threat to public health. The effective therapeutics are in urgent need. Here, we developed a high-content screening for the antiviral candidates using fluorescence-based SARS-CoV-2 nucleoprotein detection in Vero E6 cells coupled with plaque reduction assay. Among 122 Thai natural products, we found that *Boesenbergia rotunda* extract and its phytochemical compound, panduratin A, exhibited the potent anti-SARS-CoV-2 activity. Treatment with *B. rotunda* extract and panduratin A after viral infection drastically suppressed SARS-CoV-2 infectivity in Vero E6 cells with IC_50_ of 3.62 μg/mL (CC_50_ = 28.06 µg/mL) and 0.81 μΜ (CC_50_ = 14.71 µM), respectively. Also, the treatment of panduratin A at the pre-entry phase inhibited SARS-CoV-2 infection with IC_50_ of 5.30 µM (CC_50_ = 43.47 µM). Our study demonstrated, for the first time, that panduratin A exerts the inhibitory effect against SARS-CoV-2 infection at both pre-entry and post-infection phases. Apart from Vero E6 cells, treatment with this compound was able to suppress viral infectivity in human airway epithelial cells. This result confirmed the potential of panduratin A as the anti-SARS-CoV-2 agent in the major target cells in human. Since *B. rotunda* is a culinary herb generally grown in China and Southeast Asia, its extract and the purified panduratin A may serve as the promising candidates for therapeutic purposes with economic advantage during COVID-19 situation.

## Introduction

In December 2019, multiple severe pneumonia cases emerged in Wuhan, Hubei, China^1^. The causative agent was identified as a novel coronavirus, which was scientifically named severe acute respiratory syndrome coronavirus 2 (SARS-CoV-2). The World Health Organization (WHO) called the disease caused by this virus as coronavirus disease 19 or COVID-19. With the vast and rapid spreading, the virus became pandemic in a short period, causing a severe outbreak in 218 countries and territories around the world. As of October, 2020, the number of confirmed cases of COVID-19 climbed above 42 million, with more than one million deaths globally^2^. This catastrophic situation highlighted the urgent need of the entire population for the effective and affordable antiviral therapeutics to fight against the dreadful disease.

SARS-CoV-2 is an enveloped, positive-sense, single-stranded RNA virus of *Coronaviridae* family. This virus was categorized as a member of *Betacoronavirus* genus alongside severe acute respiratory syndrome coronavirus (SARS-CoV) and Middle East respiratory syndrome coronavirus (MERS-CoV). Usually, most human cases of coronavirus infection are mild or asymptomatic. However, the outbreak of SARS-CoV in 2003^3,4^. MERS-CoV in 2014^5^, and SARS-CoV-2 rang the alarm bell of the global public health crisis. Currently, there are no specific drugs for the treatment of COVID-19. All drug options are based on the treatment of the related viruses, such as SARS-CoV, MERS-CoV, influenza virus, Ebola virus, and HIV-1. Accordingly, several FDA-approved drugs with a broad therapeutic window serve as potential candidates for COVID-19 treatment^6,7^. The most promising repurposed drugs included chloroquine/hydroxychloroquine^8–10^, favipiravir^11^, lopinavir/ritonavir^12^, and remdesivir^13,14^. However, the degree of efficacy and the severe side effects of these drugs were still under controversy^12,15,16^. Apart from FDA-approved drugs, natural product-based medicines are gained much attention. The use of Thai traditional herbs, particularly their phytochemicals, has been reported to exert broad-spectrum activities as the anticancer, anti-inflammatory, antioxidant therapeutics, and antivirals^17–20^. This suggests their potential as the anti-SARS-CoV-2 candidates.

Phytochemicals and plant-derived extracts are ideal places to find a promising drug component against coronavirus^21^. Several phytochemicals are currently under investigation for their applications in treating SARS-CoV-2, as many research groups have recently reported their studies on the potential use of these materials. One of the studies led by Jin Z. et al*.*^22^, demonstrated that the main protease (M^pro^) of SARS-CoV-2, a prospective drug target involved in the viral replication and transcription, can be targeted by Shikonin, a common plant-derived naphthoquinone. Further study on the molecular docking showed a reasonable docking pose indicating that Shikonin could bind to the substrate pocket^22^. Khan et al*.*^23^, employed the computational based methods to identify chymotrypsin-like protease inhibitors (3CL^Pro^) from FDA-approved antivirals and natural compounds library. Three antiviral drugs (Remdesivir, Saquinavir, and Darunavir) and two natural compounds (flavone and coumarin derivatives) were identified as potential inhibitors for 3CL^Pro^ of the coronavirus. Another study on the structure of SARS-CoV-2 3CL^Pro^ has revealed several potential phytochemical flavonoids, including myricitrin and licoleafol, as inhibitors against this enzyme using the predicted 3D structure^24^. Although these results are encouraging, there are not enough in vitro data to further confirm the benefit and potential of these materials.

In recent years, cell-based phenotypic methods combining with high-content imaging technology have dramatically changed the landscape of the drug discovery process. This technique has proven to be valuable and influential in discovering molecules with desired biological functions in a relevant cell-based setting^25^. Due to the urgent scenario and the prospective potential of phytochemicals as an alternative treatment against novel coronavirus as demonstrated by a relevant study on their anti-SARS-CoV activities^21,26^, it has prompted us to develop, for the first time, a high-content screening platform to investigate the in vitro potential of locally obtained natural extracts and compounds found in Thai medicinal plants against SARS-CoV-2.

From this rationale, we established a high-content screening platform for the antiviral drug candidates by using a fluorescence-based technique in a standard cell line for coronavirus infection, Vero E6. A total of 122 of the extracts and purified compounds derived Thai medicinal plants were screened. The extracts and compounds with the high antiviral potency were further evaluated by dose–response analysis and plaque reduction assay. Additionally, to confirm the efficiency of the selected compound in human airway, we proved the anti-SARS-CoV-2 potential with a high-content imaging technique in human lung epithelial cells in comparison to remdesivir, the first COVID-19 drug approved by FDA. In the end, this study demonstrated that *Boesenbergia rotunda* (fingerroot) extract and its phytochemical, panduratin A, were the promising candidates for a novel treatment against COVID-19.

## Results

### High-content screening of Thai natural compounds reveals four candidates with potential anti-SARS-CoV-2 activities

**Figure 1:**
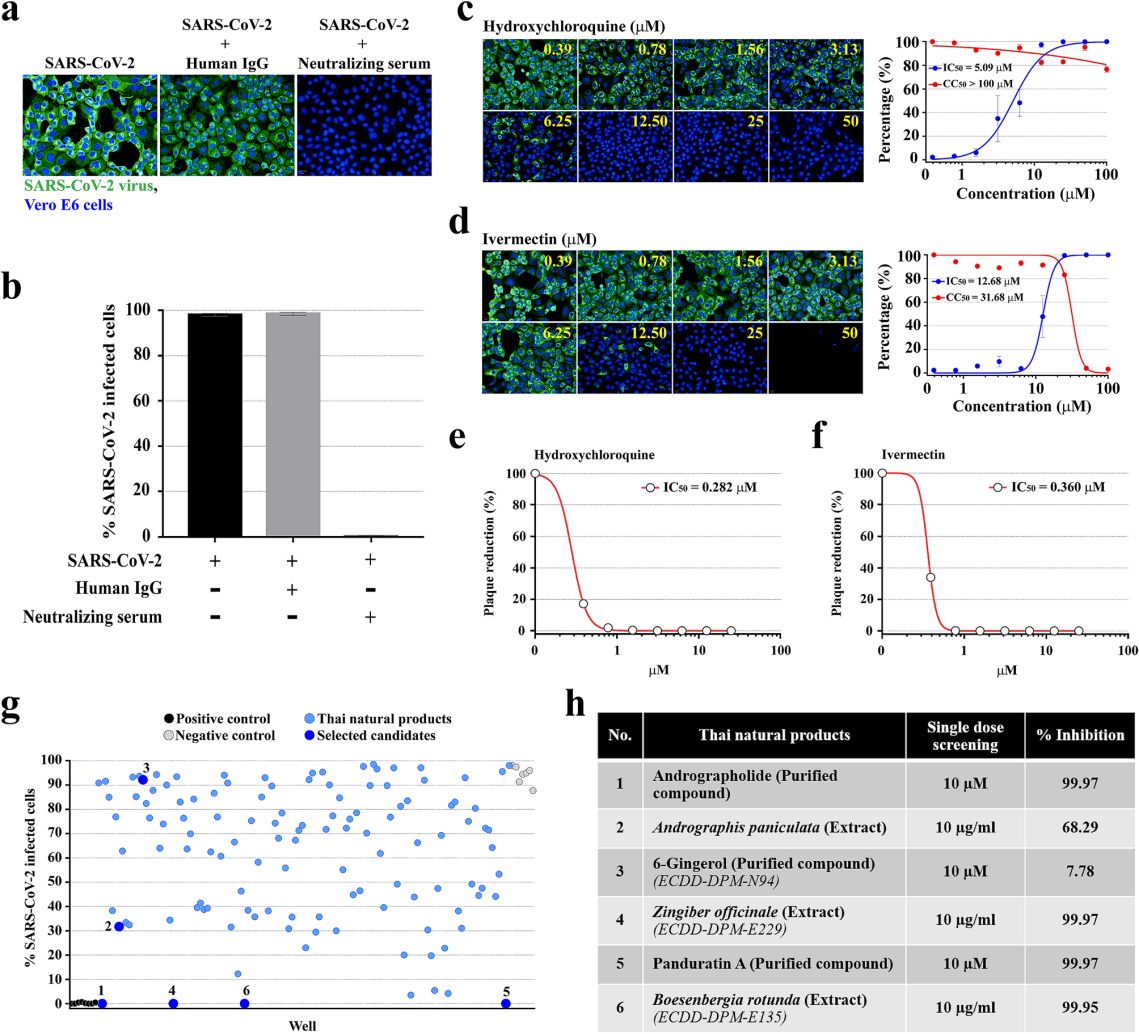
High-content anti-SARS-CoV-2 compound screening. (**a**) The SARS-CoV-2 (at 25TCID_50_) infected Vero E6 cells were detected by high-content imaging of the control condition. Fluorescent signals: green, anti-SARS-CoV NP mAb; blue, Hoechst. (**b**) Percentage of the infected Vero E6 of the control conditions. (**c**, **d**) The high-content images of the infected Vero E6 cells treated with hydroxychloroquine (**c**) and ivermectin (**d**) (the left panel). The percentage of virus inhibition (blue) and cell viability (red) was shown in the right panel (n = 3 biological replicates). (**e**, **f**) The production of infectious SARS-CoV-2 in Vero E6 cells was evaluated by plaque reduction assay after 48 h of hydroxychloroquine (**e**) and ivermectin (**f**) treatment (n = 2 biological replicates) (**g**) A total of 122 Thai natural products (114 medicinal plant extracts and 8 purified compounds) were screened for anti-SARS-CoV-2 activity (n = 2 technical replicates). (**h**) Percentage of virus inhibition of six selected candidates corresponding to the number-labeled blue dots in (**g**). Full details of the screening results provided in Supplementary Table S1.

The high-content imaging screening system was developed and optimized in Vero E6 cells infected with SARS-CoV-2 at 25TCID_50_. At 48 h after infection, the infected cells were evaluated by fluorescence analysis with the primary antibody specific to NP of SARS-CoV, which was able to cross-react with NP protein of SARS-CoV-2. The neutralizing serum from COVID-19 patient (the positive control) completely blocked SARS-CoV-2 infectivity (Fig. 1a,b). In addition, hydroxychloroquine and ivermectin, two FDA-approved drugs with reported anti-SARS-CoV2 activities in vitro and in clinical trials^8–10,28,29^, were included as reference drugs to validate our screening system. Hydroxychloroquine showed a potent antiviral effect against SARS-CoV-2 with IC_50_ of 5.08 µM. Besides, this drug had less cytotoxic with CC_50_ > 100 µM (Fig. 1c). Ivermectin demonstrated the anti-SARS-CoV-2 activity with IC_50_ of 12.68 µM. However, its therapeutic window was narrow with CC_50_ of 31.68 µM (Fig. 1d). The production of the infectious virion, as measured by plaque reduction assay, confirmed SARS-CoV-2 suppression following hydroxychloroquine and ivermectin treatments (Fig. 1e,f). This finding pointed out the high efficacy of hydroxychloroquine in the inhibition of SARS-CoV-2 infectivity in Vero E6 cells and encouraged to use this drug as the validated control in further experiments.

Subsequently, we performed the high-content screening of Thai natural products, consisting of medicinal plant extracts and phytochemical compounds, to search for the new and promising anti-SARs-CoV-2 candidates. A total of 122 of the crude extracts and the purified compounds derived from Thai natural products were investigated. Four candidates consisting of two extracts (at 10 μg/mL) of *Boesenbergia rotunda* (fingerroot) and *Zingiber officinale* (ginger), and two purified compounds (at 10 μM), i.e., andrographolide and panduratin A, exhibited 99.9% inhibitory activities against SARS-CoV-2 (Fig. 1g,h)*.* Interestingly, panduratin A is the purified compound derived from *B. rotunda.* This finding encouraged us to look for *Andrographis paniculata* and 6-Gingerol, the extract and the purified compound counterpart of andrographolide and *Z. officinale,* respectively. We found that *A. paniculata* extract (at 10 μg/mL) had moderate inhibitory activity, while 6-Gingerol (at 10 μM) had a mild effect against SARS-CoV-2 infection (Fig. 1g,h). This result suggested further evaluation of these medicinal plant extracts and phytochemical compounds in a dose–response manner.

### Dose–response relationship of six selected candidates at post-infectious phase

**Figure 2:**
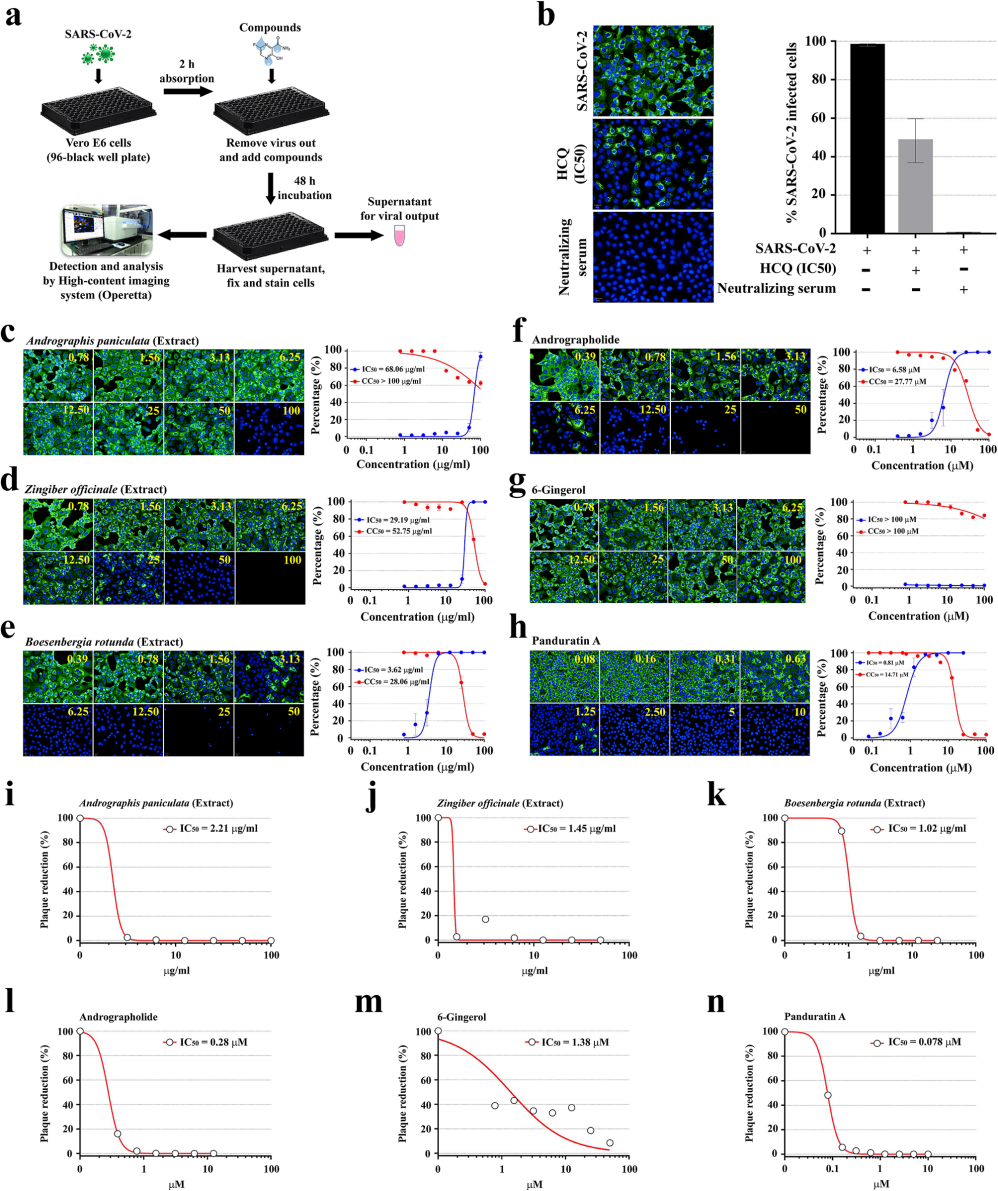
Dose-dependent anti-SARS-CoV-2 effects of six candidates at the post-infectious phase. (**a**) Study design. SARS-CoV-2 infected Vero E6 cells (at 25TCID_50_) were treated with the extract/compound for 48 h before harvest. (**b**) Controls. Hydroxychloroquine (HCQ) at the IC_50_ (5.08 µM) for post-infection treatment (from Fig. 1c) and the neutralizing serum served as the positive controls. (**c**–**h**) High-content imaging analysis of *Andrographis paniculata* extract (**c**), *Zingiber officinale* extract (**d**), *Boesenbergia rotunda* extract (**e**), Andrographolide (**f**), 6-Gingerol (**g**), and panduratin A (**h**) was demonstrated in the left panel. The percentage of virus inhibition (blue) and cell viability (red) was shown in the right panel (n = 3 biological replicates). Fluorescent signals: green, anti-SARS-CoV-2 NP mAb; blue, Hoechst. (**i**–**n**) Plaque reduction assay of six candidates, i.e., *A. paniculata* extract (**i**), *Z. officinale* extract (**j**), *B. rotunda* extract (**k**), Andrographolide (**l**), 6-Gingerol (**m**), and panduratin A (**n**) (n = 2 biological replicates).

From the initial screening, three pairs of Thai medicinal plant extracts and their purified compounds (Fig. 1g,h) were selected to further examine for antiviral potentials. In this part, the post-treatment approach was followed, in which two-fold dilutions of the extracts or the compounds were added into the cell culture after 2 h of viral adsorption and maintained for the 48 h period. Thereafter, the culture supernatants were harvested, and the cells were fixed and stained with anti-SARS-CoV NP mAb and Alexa Fluor 488-labeled secondary antibody (Fig. 2a). Hydroxychloroquine at the IC_50_ concentration (5.08 µM; as showed in Fig. 1c), together with the neutralizing serum, served as the positive controls of the experiment (Fig. 2b). Overall, each of six candidates exhibited a dose–response relationship. The extract of *A. paniculata* and its purified compound, andrographolide, showed the potent antiviral effect with IC_50_ of 68.06 µg/mL (CC_50_ > 100 µg/mL) and 6.58 µM (CC_50_ = 27.77 µM), respectively (Fig. 2c,f). The anti-SARS-CoV-2 potential of *Z. officinale* extract exhibited IC_50_ of 29.19 µg/mL (CC_50_ = 52.75 µg/mL) (Fig. 2d); however, its purified compound 6-Gingerol had lower potency with IC_50_ > 100 µM (CC_50_ > 100 µM) (Fig. 2g). Among six selected candidates, the *B. rotunda* extract and its purified compound, panduratin A, exhibited very potent anti-SARS-CoV-2 activity with IC_50_ of 3.62 µg/mL (CC_50_ = 28.06 µg/mL) and 0.81 µM (CC_50_ = 14.71 µM), respectively (Fig. 2e,h). Analyses of viral output by plaque assay (Fig. 2i–n) were consistent with the high-content screening results (Fig. 2c–h). The absolute inhibition of the infectious virion production in the post-treatment approach was observed in *A. paniculata* extract (100 µg/mL)*,* andrographolide (12.5 μM), *B. rotunda* extract (12.5 µg/mL), and panduratin A (5 µM). Collectively, *B. rotunda* extract and its purified compound panduratin A had higher anti-SARS-CoV-2 activities than other candidates.

### Anti-SARS-CoV-2 effect of *Boesenbergia rotunda* extract and panduratin A at the pre-entry phase

**Figure 3:**
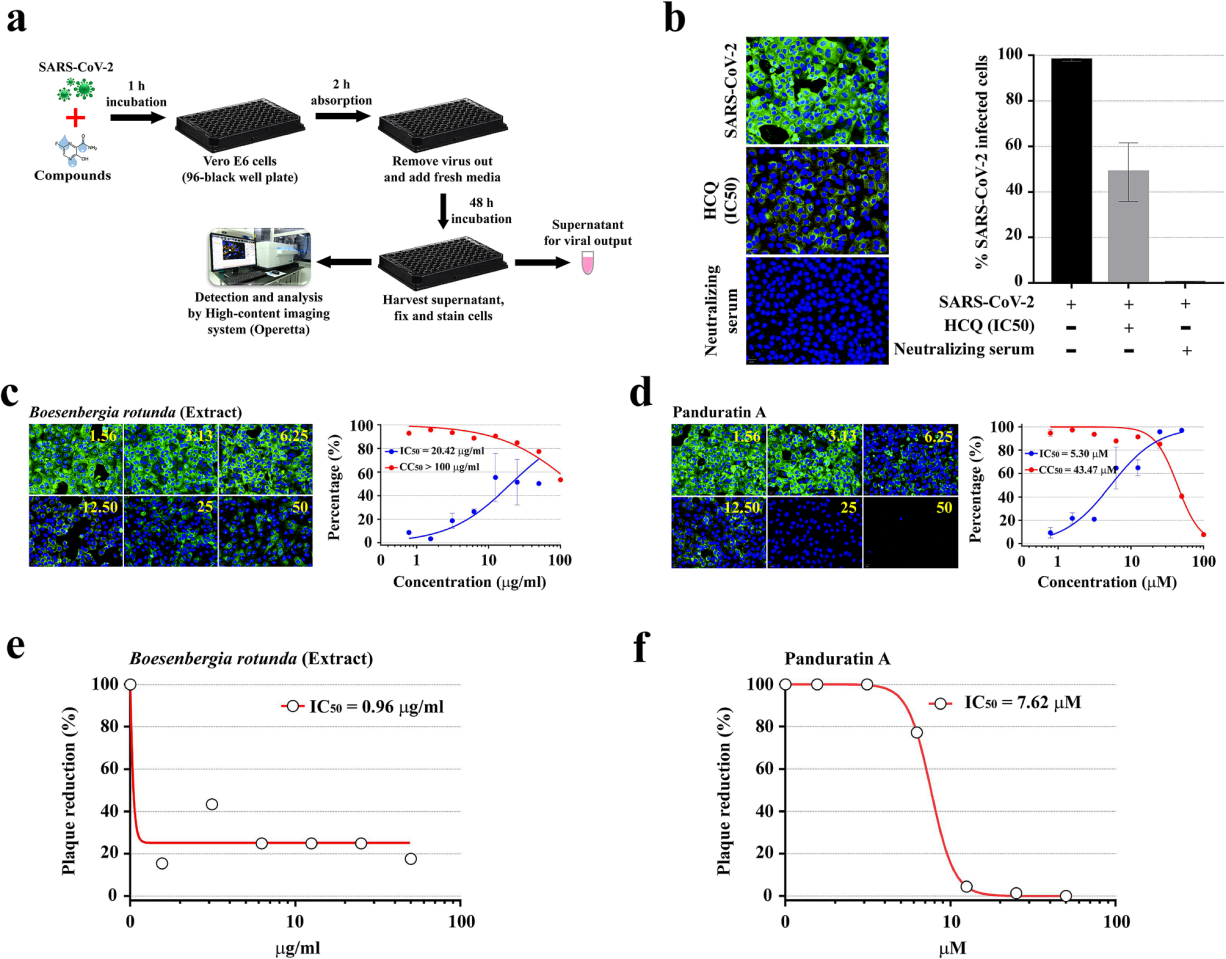
Dose-dependent anti-SARS-CoV-2 effects of *B. rotunda* extract and panduratin A at the pre-entry phase**.** (**a**) Study design. SARS-CoV-2 at 25TCID_50_ were incubated with the extract/compound for 1 h before inoculation into Vero E6 cells. Viral adsorption was allowed for 2 h in the presence of the extract/compound. After washing, the culture was maintained in fresh media for 48 h before harvest. (**b**) Controls. Hydroxychloroquine (HCQ) at the IC_50_ (8.07 µM) for pre-entry treatment (details in Supplementary Fig. 1) and the neutralizing serum served as the positive controls (n = 3 biological replicates). (**c**,**d**) High-content imaging analysis of *Boesenbergia rotunda* extract (**c**) and Panduratin A (**d**) (the left panel). The percentage of virus inhibition (blue) and cell viability (red) was shown in the right panel) (n = 3 biological replicates). Fluorescent signals: green, anti-SARS-CoV-2 NP mAb; blue, Hoechst. (**e**, **f**) Plaque reduction assay of *B. rotunda* extract (**e**) and panduratin A (**f**).

*B. rotunda* extract and panduratin A had very potent anti-SARS-CoV-2 activities in the post-infection phase. To extend this impact, it was interesting to know whether *B. rotunda* extract and panduratin A also interfere with the viral entry. Pre-entry treatment was carried out to address this issue (Fig. 3a). In this procedure, *B. rotunda* extract and panduratin A were pre-incubated with SARS-CoV-2 at 37 °C for 1 h before inoculation into Vero E6 cells. Viral adsorption was allowed for 2 h in the presence of the extract/compound. Then, the cells were washed by fresh medium to remove both the unbound viral particles and the extract/compound. Fresh medium was supplemented and the cells were further cultured for 48 h before harvest (Fig. 3a). Hydroxychloroquine (at the IC_50_ = 8.07 µM for pre-entry treatment; details in Supplementary Fig. 1) and the neutralizing serum were used as the control to validate the feasibility and interpretability of the pre-entry treatment (Fig. 3b). Interestingly, *B. rotunda* extract and panduratin A also exhibited anti-SARS-CoV-2 activities in the pre-entry phase. The IC_50_ of *B. rotunda* extract and panduratin A were 20.42 µg/mL (CC_50_ > 100 µg/mL) and 5.30 µM (CC_50_ = 43.47 µM), respectively (Fig. 3c,d). Even though it was less effective than that of post-infection condition, viral output analysis demonstrated approximately five-fold reduction of the infectious virion production following treatment with *B. rotunda* extract (Fig. 3e). Again, panduratin A absolutely suppressed the infectious virion production at a high dose of 50 μM (Fig. 3f).

We also explored whether *B. rotunda* extract and panduratin A could induce the antiviral state of the cells by treating the extract/compound with the cells before viral adsorption (Supplementary Fig. 2). The results showed the same trend even though both *B. rotunda* extract and panduratin A did not exerted a dramatic antiviral effect in this mode.

### Anti-SARS-CoV-2 effect of panduratin A in human airway epithelial cells at the post-infectious phase

**Figure 4:**
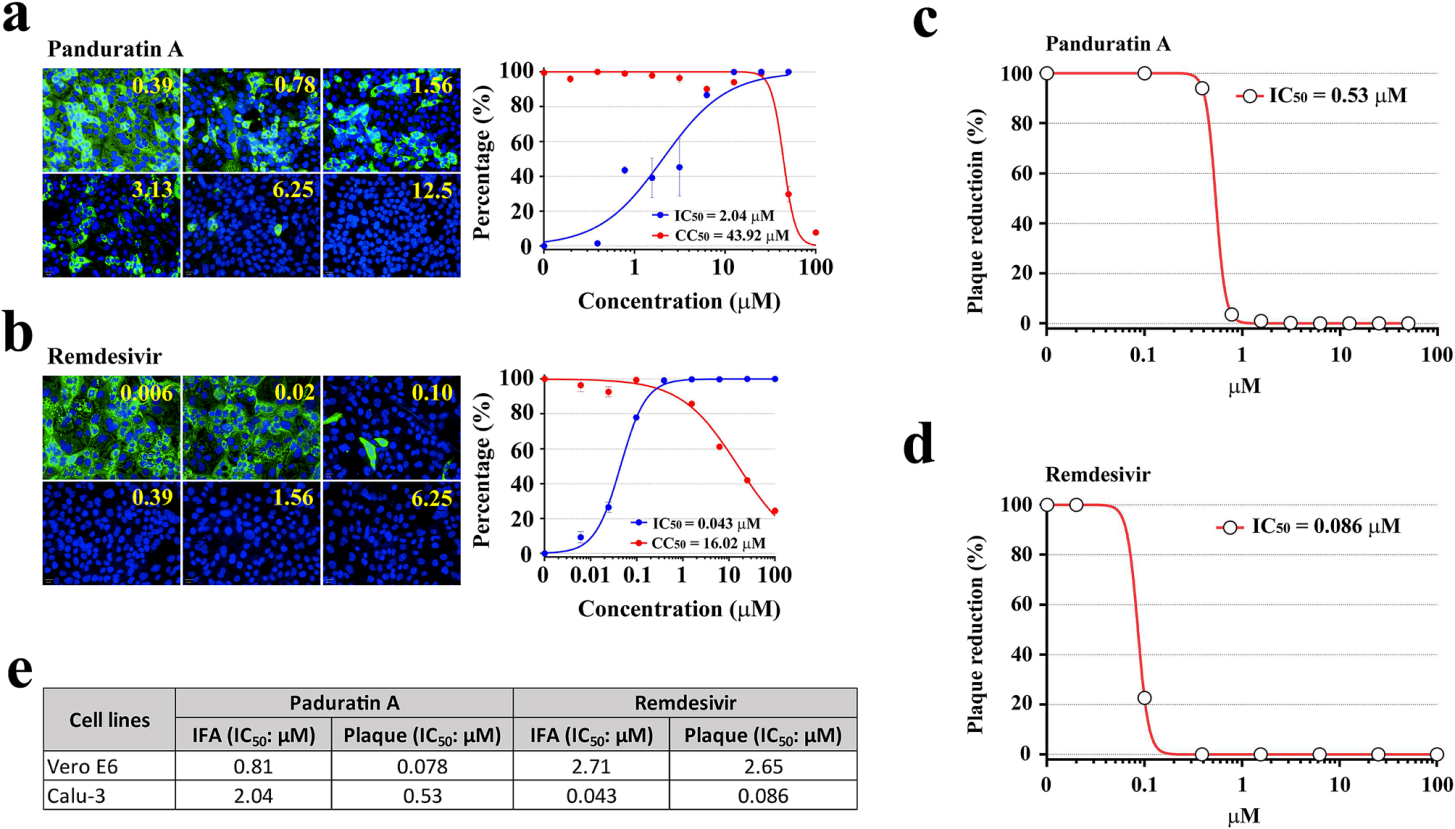
Dose-dependent anti-SARS-CoV-2 effects of panduratin A and remdesivir in human airway epithelial cells (Calu-3) at the post-entry phase. (**a**) High-content imaging analysis of panduratin A (**a**) and remdesivir (**b**) (the left panel). The percentage of virus inhibition (blue) and cell viability (red) was shown in the right panel (n = 3 biological replicates). Fluorescent signals: green, anti-SARS-CoV-2 NP mAb; blue, Hoechst. (**c**, **d**) Plaque reduction assay of panduratin A (**c**) remdesivir (**d**). (**e**) Comparison of IC_50_ values of panduration A and remdesivir evaluated by IFA of the high-content imaging technique and plaque assay in two cell types, Vero E6 and Calu-3.

Among 122 of the crude extracts and purified compounds derived from Thai natural products that were initially screened in this study, we reported panduratin A, a phytochemical compound derived from *B. rotunda*, as the most potent anti-SARS-CoV-2 agent. To prove its efficacy in the major target cells in human, Calu-3, human airway epithelial cell line, was used in the high-content imaging procedure. In the preliminary study, we evaluated that Calu-3 cells were susceptible and permissive to SARS-CoV-2, similar to Vero E6. The infectivity in Calu-3 was almost reached 100% with the high numbers of the infectious virions produced, approximately 10^7^ PFU/mL at 48 h post-infection (data not shown). In post-treatment approach, the anti-SARS-CoV-2 activity of panduratin A in Calu-3 cells was detected with IC_50_ value of 2.04 μM (CC_50_ = 43.92 μM) (Fig. 4a), in comparison to IC_50_ of 0.81 μM (CC_50_ = 14.71 μM) obtained in Vero E6 cells (Figs. 2h and 4e). Analysis of the infectious virion production by plaque assay also confirmed viral suppression upon panduratin A treatment (IC_50_ = 0.53 μM) (Fig. 4c). Additionally, in this experiment we used remdesivir as the therapeutic control at post-infectious phase (Fig. 4b). From the high-content imaging analysis, remdesivir exhibited the strong antiviral effect in Calu-3 with IC_50_ of 0.043 μM (CC_50_ = 16.02 μM). Consistent result was observed through the analysis of viral output by plaque assay (Fig. 4d) that showed IC_50_ value of 0.086 μM upon remdesivir treatment. Accordingly, this experiment confirmed the potency of panduratin A in human airway epithelial cells, which suggested the efficacy of this compound as the novel natural product-derived agent against SARS-CoV-2.

## Discussion

In this study, the high-content imaging system, coupled with the plaque assay, was utilized for the first time to identify anti-SARS-CoV-2 agents from the Thai medicinal plant library, consisting of 114 medicinal plant extracts and 8 purified compounds (details in Supplementary Table 1). Among the positive hits, the crude extract of *B. rotunda* and its purified compound, panduratin A, demonstrated the most potent inhibitory effect against SARS-CoV-2 replication and infectivity with the favorable cytotoxicity profile in Vero E6 cells. Interestingly, panduratin A inhibited SARS-CoV-2 infectivity and replication at both pre-entry and post-infection phases, and its antiviral activity was even more potent than hydroxychloroquine FDA-approved drug currently used for COVID-19 treatment^8–10^. The IC_50_, CC_50_, and the selectivity index of panduratin A and hydroxychloroquine were summarized in Table 1. Apart from the standard cell line widely used as the model for coronavirus infection, we also investigated the anti-SARS-CoV-2 effect of panduratin A in human airway epithelial cells (Calu-3). These cells represent the major targets of the virus in human. We found that panduratin A exerted the high inhibitory efficacy similar to remdesivir, a therapeutic drug firstly approved by FDA. Additionally, this compound was demonstrated to have no or minimal cytotoxicity at the therapeutic window in various cell types, including human liver cancer cell line (HepG2), immortalized hepatocyte-like cell line (imHC), human normal kidney (HK-2), human neuroblastoma cell line (SH-SY5Y), and human colon cancer cell line (Caco-2) (Supplementary Fig. 3). This finding highlighted the potential implication of panduratin A as the novel anti-SARS-CoV-2 candidate for COVID-19 therapy. Nevertheless, in vivo study and the clinical trial are needed to assess the pharmacokinetic effect and the appropriate human dose of panduratin A before clinical use.

**Table 1:** A summary of anti-SARS-CoV-2 activity (IC_50_), cytotoxicity (CC_50_), and the selectivity index (SI) of panduratin A and hydroxychloroquine in Vero E6 cells.

	IC_50_ (μM)	CC_50_ (μM)	SI (CC_50_/IC_50_)
**Post-infection**			
Panduratin A	0.81	14.71	18.16
Hydroxychloroquine	5.08	> 100	> 19.68
**Pre-entry**			
Panduratin A	5.30	43.47	8.20
Hydroxychloroquine	8.07	> 100	> 12.39

*Boesenbergia rotunda* (fingerroot) belongs to the ginger family (Z*ingiberaceae*). This herb is widely used culinarily in China and Southeast Asia. Extracts of fingerroot rhizomes are well-known for its various pharmacological effects such as anti-allergic^30^, antibacterial^31,32^, antioxidant^33^, and anti-tumor activities^34,35^. Among the major active ingredients found in fingerroot, panduratin A, a prenylated cyclohexenyl chalcone, has been reported to possibly exhibit the antiviral activity against HIV-1 and dengue virus (DENV)^36–39^.

Several molecular and cellular mechanisms might be employed by panduratin A to exert its effect on anti-SARS-CoV-2 activity. Using the biochemical approach, this phytochemical was demonstrated to physically bind and inhibit an HIV-1 protease^36^ and a DENV NS2B/NS3 protease^37^. Also, the structure-based computational approach supported panduratin A potential as the competitive inhibitor of NS2B/NS3 of DENV2^38,39^. Whether this compound interacts with those proteases in vivo is yet to be determined. In this view, panduratin A might act as the protease inhibitor to exhibit the anti-SARS-CoV-2 effect.

Another possible mechanism of panduratin A action might have occurred through its antioxidant activity. This compound itself is a potent reducing agent and can decrease levels of reactive oxygen species (ROS) in vitro^40,41^. Whether the ROS scavenging mechanism facilitates the attenuation of SARS-CoV-2 infection by panduratin A, similar to that observed in Japanese Encephalitis virus (JEV)^42^, is yet to be deciphered. Further, this anti-oxidative stress might be coupled with anti-inflammatory responses widely reported for panduratin A. For example, panduratin A can reduce the expression of genes whose function is involved in inflammation^43–46^. Undoubtedly, therapeutic strategies aiming at the modulation of inflammation has been proposed for COVID-19 as a mean to reduce the severity of the disease^47^.

Besides, panduratin A was found to induce autophagy, which is vital in restricting viral replication. Nonetheless, concerns have also been raised regarding the protective role of autophagy for the evasion of host innate immunity upon viral infection^48–50^. Autophagic induction by panduratin A treatment in mammalian cells occurred through the activation of AMPK and inhibition of mTORC1^51,52^. The small molecule compound has also been shown to induce PERK/eIF2α/ATF4/CHOP pathway pertinent to endoplasmic reticulum (ER) stress. Consequently, the induction of ER stress can further facilitate autophagy^53,54^. Moreover, panduratin A can stimulate AMPK signaling leading to the activation of PPARα and PPARδ^55,56^. The induction of these transcription factor machinery can, in turn, promote autophagy^57,58^. Consistently, it was reported that MERS-CoV blocked the fusion of autophagosomes and lysosomes. As a result, the induction of autophagy attenuated the replication of this virus^59^. Interestingly, the anti-helminthic and FDA-approved drug niclosamide has recently been proposed as a potential anti-SARS-CoV-2 agent^60,61^, possibly through its autophagic induction mechanism^62^. It has yet to be elucidated whether panduratin A suppresses SARS-CoV-2 infection via the induction of autophagy, and which pathway is a direct target for this compound.

Taken together, we identified *B. rotunda* extract and its active compound, panduratin A, as the promising anti-SARS-CoV-2 agents by using the high-content imaging system coupled with the plaque reduction assay. Importantly, *B. rotunda* extract and panduratin A exhibited the potent antiviral efficacy in Vero E6 cells when the treatment was performed after SARS-CoV-2 infection, with the optimal IC_50_ (3.62 μg/mL and 0.81 μM, respectively) and the favorable cytotoxicity profile (CC_50_ 28.06 μg/mL and 14.71 μM, respectively). Panduratin A inhibited SARS-CoV-2 infectivity in the pre-entry phase as well. The information from this present study suggested the promise of panduratin A as a single therapy, and as the combinational therapeutic with other FDA-approved agents, for the effective treatment of COVID-19. The possibility of this rationale should be further evaluated. Since *B. rotunda* is the common plant affordable and available in tropical regions, a pharmaceutically active compound derived from *B. rotunda* offers a tremendous therapeutic opportunity to fight in this bloody COVID-19 battlefield. Accordingly, we suggested panduratin A as the novel natural candidate for anti-SARS-CoV-2 infection.

## Materials and methods

### Study design

In this in vitro phenotypic screening of medicinal plant extracts and phytochemicals, the experiments were performed in two approaches; pre-entry and post-infectious treatments.

The pre-entry condition was designed based on the hypothesis that a particular extract or compound could participate in direct interaction with virion and hinder viral entry into the target cells. The drugs, natural extracts, or phytochemicals were pre-incubated with the virus before the inoculation of the mixture into the cells.

For the post-treatment, this approach aimed to investigate the effect of the selected drugs, natural extract, or phytochemicals in the ability to inhibit SARS-CoV-2 infectivity once the viral adsorption has been initiated. From this rationale, the drugs, natural extracts, or phytochemicals were supplemented into the culture medium after viral infection and maintained throughout the experimental period.

### Cell culture

Vero E6 cells, African green monkey (*Cercopithecus aethiops*) kidney epithelial cells (ATCC, USA), were initially used for the antiviral screening in this study. The cells were grown in Dulbecco’s Modified Eagle Medium (DMEM) (Gibco, USA) with 10% fetal bovine serum (FBS) (Gibco, USA). For Vero cells (African green monkey epithelial cells), these cells were cultured in Minimum Essential Medium (MEM) (Gibco, USA) supplemented with 10% FBS and L-glutamine (Gibco, USA).

Human airway epithelial cell line (Calu-3) was obtained from American Type Culture Collection (ATCC, USA). The cells were maintained in Dulbecco's Modified Eagle Medium: Nutrient Mixture F-12 (DMEM/F-12) (Gibco, USA). supplemented with 10% fetal bovine serum (FBS) (Thermo Scientific Fisher, USA), 100 μg/ml penicillin/streptomycin (Invitrogen, USA) and 1% GlutaMAX (Gibco, USA). All cultures were grown at 37 °C in a humidified incubator with 5% CO_2_.

### Virus

SARS-CoV-2 virus (SARS-CoV-2/01/human/Jan2020/Thailand) was isolated from nasopharyngeal swabs of a confirmed COVID-19 patient in Thailand. The virus was propagated in Vero E6 cells by three passages to establish a high-titer stock (passage 4) and stored at − 80 °C for using in all experiments. Virus titration as TCID_50_ titer/mL was performed in the 96-well microtiter plate. Briefly, the virus stock was titrated in quadruplicate in 96-well microtiter plates on Vero E6 cells in serial dilution to obtain 50% tissue culture infectious dose (TCID_50_) by using the Reed Muench method^27^. All the experiments with live SARS-CoV-2 virus were performed at a certified biosafety level 3 facility.

### Plant materials

Plant materials in the screening study were common herbs in Thailand, and most of them were listed in Thai Herbal Pharmacopoeia 2018 (https://bdn.go.th/th/sDetail/10/34/). *Boesenbergia rotunda* rhizomes were purchased from suppliers in Pathum Thani, Thailand. The plant was identified and compared with depository plant materials of ECDD before starting extraction procedures.

### Extracts and compounds

The air-dried and finely powdered rhizomes of *B. rotunda* (2.5 kg) were percolated with 95% EtOH (6 L, 4 times × 7 days) at room temperature to give a crude EtOH extract (190.5 g) after solvent removal. The obtained EtOH extract was divided into two portions. Each portion was separated by VLC over Si-gel (250 g each, Merck Art. No. 7731), packing on a sintered glass funnel (i.d. 12.5 cm × packing height 4.5 cm), using EtOAc-hexanes and MeOH-EtOAc gradients as eluents, respectively. Fractions (500 mL each) were collected and combined based on their TLC behaviors to give frs. A_1_–A_5_. Fr. A_4_ (60.1 g, eluted with 25–100% EtOAc-hexanes), after three further consecutive Si-gel CC (Si-gel: Merck, Art. No 7734, 1st CC: 20% EtOAc-hexanes; 2nd CC: 60% CH_2_Cl_2_-hexanes; 3rd CC: 10% CH_3_COCH_3_-hexanes) afforded three separated frs. B_1_–B_3_. Fr. B_3_ (5.37 g) was further purified by Sephadex LH-20 CC (Sephadex LH-20: GE Healthcare Bio-Sciences AB, 10% MeOH-CH_2_Cl_2_), followed by recrystallization from EtOH-CH_2_Cl_2_ to provide pure panduratin A (3.18 g).

### In vitro antiviral assay

A total of 1 × 10^4^ Vero E6 cells were cultured in a 96-black well plate (Corning, USA) for 24 h at 37 °C in 5% CO_2_ atmosphere. Then, culture supernatant was discarded, and the cells were washed once with phosphate-buffered saline (PBS). In the case of post-treatment, the cells were subsequently infected with SARS-CoV-2 at 25TCID_50_. After viral adsorption for 2 h at 37 °C, the cells were washed twice to remove the excessive inoculum with PBS, and the fresh culture medium (DMEM with 2% FBS) was added into the wells. Each concentration of drugs, crude extracts, or active compounds was directly inoculated into the culture medium. The cells were then maintained at 37 °C in 5% CO_2_ incubator for 48 h. For pre-entry treatment, the mixture of each drug, crude extract, or active compound and 25TCID_50_ of SARS-CoV-2 was incubated at 37 °C for 1 h before inoculating it into the cells. Similarly, viral adsorption was allowed for 2 h. After that, the cells were washed twice with PBS, and the fresh culture medium (DMEM with 2% FBS) was added into the cells. The culture was maintained for an additional 48 h. For the pre-treatment experiment, each concentration of drugs, crude extracts, or active compounds was directly inoculated into the cells before viral infection. After incubation at 37 °C for 1 h, drug, crude extract or compound was removed and the cells were washed with PBS. Then, the cells were infected with SARS-CoV-2 at 25TCID_50_. Viral adsorption was carried out for 2 h at 37 °C followed by washing with PBS. Fresh medium (DMEM with 2% FBS) was added into the wells, and the culture was maintained for 48 h. Positive convalescent serum (heat-inactivated at 56 °C for 30 min.) of a COVID-19 patient and anti-human IgG-FITC (Santa Cruz Biotechnology, USA) was used as a viral inhibition positive control and negative control, respectively. The experiment was done in triplicate.

### High-content imaging system for SARS-CoV nucleoprotein detection

In each treatment condition, the cells in the 96-well plate were fixed and permeabilized with 50% (v/v) acetone in methanol on ice for 20 min. The cells were washed once with phosphate-buffered saline with 0.5% Tween detergent (PBST) and blocked in PBST with 2% (w/v) BSA for 1 h at room temperature. After blocking, the cells were incubated with 1:500 dilution ratio of primary antibody specific for SARS-CoV Nucleoprotein (NP) (Rabbit mAb) (Sino Biological Inc., China) for 1 h at 37 °C. This antibody can cross-react with the NP protein of SARS-CoV-2 as well. The unbound antibody was removed by washing with PBST three times. Then, the Goat anti-Rabbit IgG (H + L) Highly Cross-Adsorbed Secondary Antibody, Alexa Fluor 488 (Thermo Fisher Scientific, USA), was used at 1:500 dilution ratio. Nuclei of the cells were stained with Hoechst dye (Thermo Fisher Scientific, USA). The fluorescent signals were detected and analyzed by the high-content imaging system (Operetta, PerkinElmer) at 40 × magnification. The percentage of the infected cells in each well was automatically obtained from 13 images per well using Harmony software (PerkinElmer, USA) (the parameters and the analytical sequence were provided in Supplementary Information).

### Plaque assay

The viral output of SARS-CoV-2 was reported as the infectious titers that were determined by plaque assay. In brief, Vero cell monolayer was seeded into 6-well plate 24 h before infection. The cells were inoculated with a serial dilution of the virus and incubated for viral adsorption for 1 h at 37 °C. Then, the cells were overlaid with 3 mL/well of overlay medium containing MEM supplemented with 5% FBS and 1% agarose. The culture was incubated at 37 °C in 5% CO_2_ for three days to allow plaque development. After that, plaque phenotypes were visualized by staining with 0.33% Neutral Red solution (Sigma-Aldrich, USA) for 5 h. Plaque numbers were counted as plaque-forming units per milliliter (PFUs/mL) and presented as the percentage of plaque reduction.

### Cell viability assay

Cells were seeded on 96-well plates at 5 × 10^4^ cells/well and treated with various concentrations of the extracts or purified compounds for 48 h. Cell viability was evaluated by an MTT colorimetric assay. In brief, the medium was replaced with MTT [3-(4,5-dimethylthiazol-2-yl)-2,5-diphenyltetrazolium bromide] (Sigma-Aldrich, USA) and incubated for 4 h at 37 °C in a humidified incubator with 5% CO_2_. The formazan crystal was dissolved with DMSO (Merck, Germany) and measured at a wavelength of 570 nm by EnVision Multilabel Reader (PerkinElmer, USA). Data was normalized versus the solvent control, and then CC_50_ values were calculated using GraphPad Prism 7.

## Supplementary information

Supplementary Information.

## Data Availability

All data generated or analyzed during this study are included in this article and Supplementary Information files.
