# Supplementary material for: High-content screening of Thai medicinal plants reveals Boesenbergia rotunda extract and its component Panduratin A as anti-SARS-CoV-2 agents
Source: Sci Rep. 2020 Nov 17;10:19963. doi: 10.1038/s41598-020-77003-3 (PMC7672115; doi:10.1038/s41598-020-77003-3)
Supplement: Supplementary file 1 — Supplementary Information. [file 41598_2020_77003_MOESM1_ESM.docx]

**High-Content Screening of Thai Medicinal Plants Reveals *Boesenbergia rotunda* Extract and its Component Panduratin A as Anti-SARS-CoV-2 Agents**

**Phongthon Kanjanasirirat**,^1,+^ **Ampa Suksatu**,^2,+^ **Suwimon Manopwisedjaroen**,^2^ **Bamroong Munyoo**,^1^ **Patoomratana Tuchinda**,^1,3^ **Kedchin Jearawuttanakul**,^1^ **Sawinee Seemakhan**,^1^ **Sitthivut Charoensutthivarakul**,^1,4,9^ **Patompon Wongtrakoongate**,^5,9^ **Noppawan Rangkasenee**,^1^ **Supaporn Pitiporn**,^7^ **Neti Waranuch**,^8^ **Napason Chabang**,^4^ **Phisit Khemawoot**,^10^ **Khanit Sa-ngiamsuntorn**,^11^ **Yongyut Pewkliang**,^12^ **Piyanoot Thongsri**,^12^ **Somchai Chutipongtanate**,^13^ **Suradej Hongeng**,^1,13^ **Suparerk Borwornpinyo**,^1,6,^* **Arunee Thitithanyanont**^2,^*

^1^ Excellence Center for Drug Discovery (ECDD), Faculty of Science, Mahidol University, Bangkok 10400 Thailand.

^2^ Department of Microbiology, Faculty of Science, Mahidol University, Bangkok 10400 Thailand.

^3^ Department of Chemistry, Faculty of Science, Mahidol University, Bangkok 10400 Thailand.

^4^ School of Bioinnovation and Bio-based Product Intelligence, Faculty of Science, Mahidol University, Bangkok 10400 Thailand.

^5^ Department of Biochemistry, Faculty of Science, Mahidol University, Bangkok 10400 Thailand.

^6^ Department of Biotechnology, Faculty of Science, Mahidol University, Bangkok 10400 Thailand.

^7^ Chao Phraya Aphaiphubet Hospital, Prachin Buri 25000 Thailand.

^8^ Department of Pharmaceutical Technology, Faculty of Pharmaceutical Sciences, Naresuan University, Phitsanulok 65000 Thailand.

^9^ Center for neuroscience, Faculty of Science, Mahidol University, Bangkok 10400 Thailand

^10^ Chakri Naruebodindra Medical Institute, Faculty of Medicine Ramathibodi Hospital, Mahidol University, Samutprakarn 10540 Thailand.

^11^ Department of Biochemistry, Faculty of Pharmacy, Mahidol University, Bangkok 10400, Thailand.

^12^ Section for Translational Medicine, Faculty of Medicine Ramathibodi Hospital, Mahidol University, Bangkok 10400, Thailand

^13^ Department of Pediatrics, Faculty of Medicine Ramathibodi Hospital, Mahidol University, Bangkok 10400 Thailand.

^+^ These authors contributed equally to this work

* Shared co-corresponding authors:

Suparerk Borwornpinyo, Ph.D.

Excellence Center for Drug Discovery (ECDD), Faculty of Science, Mahidol University, Bangkok 10400 Thailand.

email: [bsuparerk@gmail.com](mailto:bsuparerk@gmail.com)

Arunee Thitithanyanont, M.D.

Department of Microbiology, Faculty of Science, Mahidol University, Bangkok 10400 Thailand.

email: [arunee.thi@mahidol.edu](mailto:arunee.thi@mahidol.edu)

**SUPPLEMENTARY INFORMATION**

**Supplementary Methods**

**High-content image analytical sequence**

Each images were analyzed by using Harmony software (PerkinElmer) with the following analytical sequence;

1. Input image – Individual planes, Flatefield Correction: Basic

2. Find Nuclei – Channel: HOECHST 33342; Method: C; Output Population: Total cells

3. Find Cytoplasm – Channel: HOECHST 33342; Nuclei: Total cells; Method: A

4. Find Spots – Channel: Alexa 488; ROI Population: Total cells; ROI Region: Cytoplasm; Method: B; Output population: Spots

5. Calculate Intensity Properties – Channel: Alexa 488; Population: Total cells; Region: Cytoplasm; Method: Standard; Output Properties: Intensity Cytoplasm Alexa 488

6. Select Population – Population: Total cells; Method: Filter by Property (Number of Spots and Intensity Cytoplasm Alexa 488); Output Population: Infected cells

7. Calculate Intensity Properties (2) – Channel: Alexa 488; Population: Infected cells; Region: Cytoplasm; Method: Standard; Output Properties: Intensity of infected cells

8. Define Results:

8.1 Method – List of outputs:

- Total cells: Number of objects

- Infected cells: number of objects

8.2 Method – Formula output:

- Formula: (100*a)/b

- Variation a: Infected cells – Number of objects

- Variation b: Total cells – Number of objects

- Output name: % Infected cells

8.3 Single cell results: All

**Supplementary Table**

**Supplementary Table 1**. A list of 122 natural extracts and compounds being evaluated by the high-content screening for the candidates of the anti-SARS-CoV-2 agents.

| **ECDD Code** | **Scientific name** | **%Inhibition at 10 μg/ml (or μM)** |
| --- | --- | --- |
| ECDD-NAM | alpha-mangostin | 9.08 |
| ECDD-NAD | Andrographolide | 99.97 |
| ECDD-EGM | Garcinia mangostana L. | 8.42 |
| ECDD-EGME | Garcinia mangostana L. | 15 |
| ECDD-EVTE | Vitex trifolia | 61.69 |
| ECDD-ECLE | Chan Leela recipe | 23.07 |
| ECDD-EAPE | Andrographis paniculata | 68.29 |
| ECDD-EAP | Andrographis paniculata | 37.15 |
| ECDD-E461103 | Andrographis paniculata | 66.54 |
| ECDD-EX630209VANN | Andrographis paniculata | 67.59 |
| ECDD-EBP-1 | Buppo-1 recipe | 6.7 |
| ECDD-EBP-2 | Buppo-2 recipe | 14.76 |
| ECDD-EBP-3 | Buppo-3 recipe | 6.32 |
| ECDD-DPM-N94 | 6-Gingerol | 7.78 |
| ECDD-DPM-N95 | 8-Gingerol | 17.59 |
| ECDD-DPM-N96 | 10-Gingerol | 23.51 |
| ECDD-DPM-N97 | 6-Shogaol | 12.17 |
| MUC1775 | 10-Shogaol | 5.7 |
| ECDD-DPM-E50 | Zingiber officinale | 35.97 |
| ECDD-DPM-E51 | Zingiber officinale | 26.02 |
| ECDD-DPM-E227 | Zingiber officinale | 9.98 |
| ECDD-DPM-E228 | Zingiber officinale | 65.58 |
| ECDD-DPM-E229 | Zingiber officinale | 99.97 |
| ECDD-DPM-E55 | Clitoria ternatea | 6.58 |
| ECDD-DPM-E116 | Allium sativum | 17 |
| ECDD-DPM-E117 | Allium ampeloprasum var. ampeloprasum | 23.62 |
| ECDD-DPM-E118 | Allium ampeloprasum var. ampeloprasum | 36.29 |
| ECDD-DPM-E119 | Allium chinense | 30.07 |
| ECDD-DPM-E120 | Allium sativum | 15.76 |
| ECDD-DPM-E121 | Allium sativum | 60.45 |
| ECDD-DPM-E122 | Allium sativum | 58.56 |
| ECDD-DPM-E123 | Honey garlic recipe | 61.23 |
| ECDD-DPM-E124 | Allium chinense | 60.67 |
| ECDD-DPM-E125 | Plukenetia volubilis | 37.49 |
| ECDD-DPM-E126 | Curcuma Longa Linn. | 13.37 |
| ECDD-DPM-E127 | Curcuma longa | 23.11 |
| ECDD-DPM-E128 | Curcuma domestica Valeton | 39.29 |
| ECDD-DPM-E129 | Kaempferia parviflora | 5.89 |
| ECDD-DPM-E130 | Plukenetia volubilis | 9.13 |
| ECDD-DPM-E131 | Helianthus tuberosus | 68.44 |
| ECDD-DPM-E132 | Zingiber montanum | 33.47 |
| ECDD-DPM-E133 | Kaempferia galanga | 87.73 |
| ECDD-DPM-E134 | Curcuma amarissima Roscoe. | 53.7 |
| ECDD-DPM-E135 | Boesenbergia rotunda | 99.95 |
| ECDD-DPM-E136 | Curcuma zanthorrhiza | 61.55 |
| ECDD-DPM-E137 | Talinum paniculatum (Jacq.) Gaertn | 24.65 |
| ECDD-DPM-E138 | Boesenbergia rotunda | 64.23 |
| ECDD-DPM-E139 | Hibiscus sabdariffa | 41.78 |
| ECDD-DPM-E140 | Curcuma longa | 14.91 |
| ECDD-DPM-E141 | Zingiber officinale | 6.95 |
| ECDD-DPM-E142 | Curcuma mangga Valeton & Zijp | 61.79 |
| ECDD-DPM-E143 | Phyllanthus emblica | 10.4 |
| ECDD-DPM-E144 | Zingiber montanum (J.Koenig) Link ex A.Dietr. | 25.66 |
| ECDD-DPM-E145 | Alpinia galanga | 31.9 |
| ECDD-DPM-E146 | Senna siamea | 21.48 |
| ECDD-DPM-E147 | Anamirta cocculus (L.) Wight & Arn. | 44.16 |
| ECDD-DPM-E148 | Carthamus tinctorius | 69.16 |
| ECDD-DPM-E149 | Derris scandens | 64.33 |
| ECDD-DPM-E150 | Tinospora cordifolia | 32.68 |
| ECDD-DPM-E151 | Gynostemma pentaphyllum | 28.73 |
| ECDD-DPM-E152 | Cissus quadrangularis | 26.58 |
| ECDD-DPM-E153 | Phum chiwa recipe | 76.95 |
| ECDD-DPM-E154 | Momordica charantia | 7.74 |
| ECDD-DPM-E155 | Moringa oleifera | 5.04 |
| ECDD-DPM-E156 | Shasthara recipe | 70.57 |
| ECDD-DPM-E157 | Murdannia loriformis | 64.29 |
| ECDD-DPM-E158 | Acanthus ebracteatus | 4.68 |
| ECDD-DPM-E159 | Centella asiatica | 28.2 |
| ECDD-DPM-E160 | Vernonia cinerea (L.) Less. | 9.92 |
| ECDD-DPM-E161 | Camellia sinensis (L.) Kuntze | 22.68 |
| ECDD-DPM-E162 | Piper nigrum | 70.03 |
| ECDD-DPM-E163 | Piper longum | 15.25 |
| ECDD-DPM-E164 | Zingiber officinale | 44.87 |
| ECDD-DPM-E165 | Ipomoea pes-caprae | 27.74 |
| ECDD-DPM-E166 | Asparagus racemosus Willd. | 24.02 |
| ECDD-DPM-E167 | Pogostemon cablin | 55.18 |
| ECDD-DPM-E168 | Aquilaria crassna | 21.42 |
| ECDD-DPM-E169 | Musa acuminata Colla | 53.46 |
| ECDD-DPM-E170 | Morus | 2.29 |
| ECDD-DPM-E171 | Artocarpus lacucha | 29.8 |
| ECDD-DPM-E172 | Terminalia chebula | 10.16 |
| ECDD-DPM-E173 | Terminalia bellirica | 1.45 |
| ECDD-DPM-E174 | Phyllanthus emblica | 3.34 |
| ECDD-DPM-E175 | Curcuma longa | 38.09 |
| ECDD-DPM-E176 | Aloe vera | 60.42 |
| ECDD-DPM-E177 | Thunbergia laurifolia | 10.19 |
| ECDD-DPM-E178 | Raphanus sativus var. Longipinnatus | 23.2 |
| ECDD-DPM-E179 | Tectona grandis | 2.89 |
| ECDD-DPM-E180 | Bombyx Mori. | 50.79 |
| ECDD-DPM-E181 | Anacardium occidentale | 18.71 |
| ECDD-DPM-E182 | Equisetidae | 79.95 |
| ECDD-DPM-E183 | Plantago major | 16.46 |
| ECDD-DPM-E184 | Cymbopogon citratus | 96.47 |
| ECDD-DPM-E185 | Polygala chinensis L. | 56.05 |
| ECDD-DPM-E186 | Senna alata | 33.73 |
| ECDD-DPM-E187 | Glycyrrhiza glabra | 2.81 |
| ECDD-DPM-E188 | Paederia pilifera Hook.f. | 7.94 |
| ECDD-DPM-E189 | Saururaceae | 69.65 |
| ECDD-DPM-E190 | Chromolaena odorata | 80.28 |
| ECDD-DPM-E191 | Gymnema inodorum Decne. | 94.59 |
| ECDD-DPM-E192 | Oroxylum indicum | 52.59 |
| ECDD-DPM-E193 | Centotheca lappacea (L.) Desv. | 30.72 |
| ECDD-DPM-E194 | Nelumbo nucifera Gaertn. | 77.16 |
| ECDD-DPM-E195 | Boesenbergia rotunda | 95.78 |
| ECDD-DPM-E196 | Gymnanthemum extensum | 18.35 |
| ECDD-DPM-E197 | Curcuma zedoaria | 17.01 |
| ECDD-DPM-E198 | Kaempferia galanga | 61.83 |
| ECDD-DPM-E199 | Cissus quadrangularis | 68.98 |
| ECDD-DPM-E200 | Momordica charantia | 6.94 |
| ECDD-DPM-E201 | Derris scandens | 24.91 |
| ECDD-DPM-E202 | Murdannia loriformis | 50.8 |
| ECDD-DPM-E203 | Gynostemma pentaphyllum | 19.58 |
| ECDD-DPM-E204 | Vernonia cinerea (L.) Less. | 55.41 |
| ECDD-DPM-E205 | Justicia gendarussa Burm.f. | 52.44 |
| ECDD-DPM-E206 | Salacia chinensis L. | 27.73 |
| ECDD-DPM-E207 | Salacia verrucosa | 28.52 |
| ECDD-DPM-E208 | Elephantopus scaber | 35.7 |
| ECDD-DPM-E209 | Rhinacanthus nasutus | 55.85 |
| ECDD-DPM-E210 | Cryptolepis dubia (Burm.f.) M.R. Almeida | 46.68 |
| ECDD-DPM-E1029 | Wan dok hemn recipe | 4.42 |
| ECDD-EEFE | Eupatorium fortunei Turcz. | 1.89 |
| ECDD-NPA | Panduratin A | 99.97 |

**Supplementary Figures**

**Supplementary Figure 1**. Dose-dependent anti-SARS-CoV-2 activities of hydroxychloroquine at the pre-entry and pre-infectious phases. **(a).** At the pre-entry phase, hydroxychloroquine had the IC_50_ of 8.07 μM and CC_50_ >100 μM. **(b).** At the pre-infection phase, hydroxychloroquine exhibited the IC_50_ of 20.55 μM and CC_50_ >100 μM.

**Supplementary Figure 2**. **Dose-dependent anti-SARS-CoV-2 effects of *B. rotunda* extract and panduratin A at the pre-infection phase. (a).** Study design. the extract/compound were incubated with Vero E6 cells for 1 hr before washing by fresh media. Thereafter, SARS-CoV-2 at 25TCID_50_ were inoculated into Vero E6 cells and viral adsorption was allowed for 2 hrs. Next, the cells were washed again to remove unbound virions and the culture was maintained in fresh media for 48 hrs before harvest. **(b)**. Controls. Hydroxychloroquine (HCQ) at the IC_50_ (20.55 µM) for pre-infection treatment (details in **Supplementary** **Figure 1b**), and the neutralizing serum served as the positive conditions (n=3 biological replicates). **(c-d).** High-content imaging analysis of *B. rotunda* extract **(c)** and Panduratin A **(d)** was demonstrated in the left panel. The percentage of virus inhibition (blue) and cell viability (red) was shown in the right panel (n=3 biological replicates). Fluorescent signals: green, anti-SARS-CoV-2 NP mAb; blue, Hoechst. **(e-f).** Plaque reduction assay of *B. rotunda* extract **(e)** and panduratin A **(f)** (n=2 biological replicates).


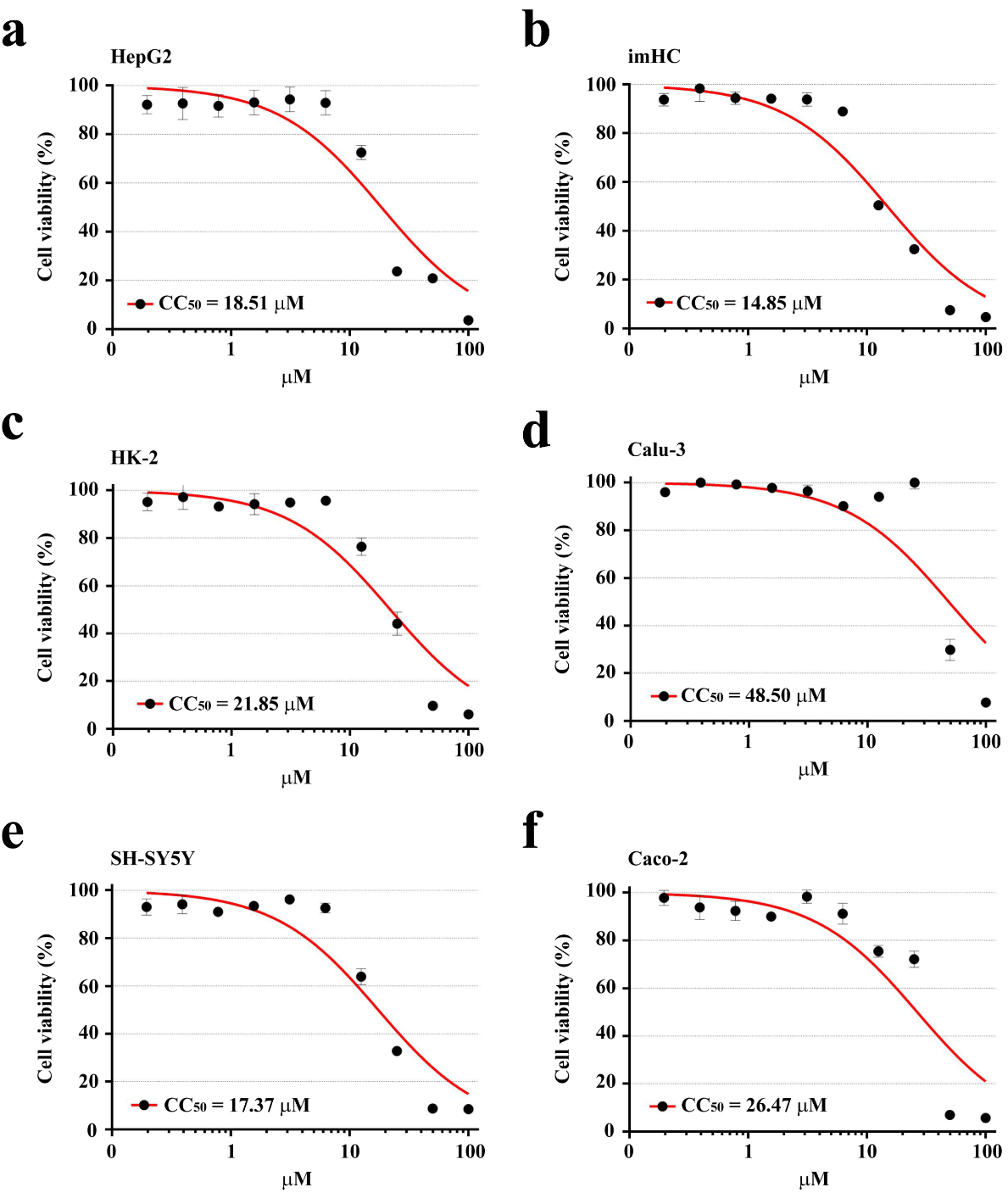


**Supplementary Figure 3**. **Percentage of cell viability upon treatment with various concentration of panduratin A.** Cells including **(a).** human liver cancer cell line (HepG2), **(b).** immortalized hepatocyte-like cell line (imHC), **(c).** human normal kidney (HK-2), **(d).** human airway epithelial cell line (Calu-3), **(e).** human neuroblastoma cell line (SH-SY5Y), and **(f).** human colon cancer cell line (Caco-2) were seeded on 96-well plates at 5 × 10^4^ cells/well and treated with various concentrations of panduratin A (0 - 100 μM) for 48 h. Cell viability was evaluated by an MTT colorimetric assay (n=3 biological replicates). Data was normalized versus the solvent control, and then CC_50_ values were calculated using GraphPad Prism 7.
